# Impact of early salmon louse, *Lepeophtheirus salmonis*, infestation and differences in survival and marine growth of sea-ranched Atlantic salmon, *Salmo salar* L., smolts 1997–2009

**DOI:** 10.1111/jfd.12052

**Published:** 2013-01-13

**Authors:** O T Skilbrei, B Finstad, K Urdal, G Bakke, F Kroglund, R Strand

**Affiliations:** 1 Institute of Marine ResearchBergen, Norway; 2 Norwegian Institute for Nature ResearchTrondheim, Norway; 3 Rådgivende Biologer ASBredsgården, Bryggen, Bergen, Norway; 4 Norwegian Institute for Water ResearchGrimstad, Norway

**Keywords:** Atlantic salmon smolt, emamectin benzoate, marine growth and survival, salmon lice, smolt releases

## Abstract

The impact of salmon lice on the survival of migrating Atlantic salmon smolts was studied by comparing the adult returns of sea-ranched smolts treated for sea lice using emamectin benzoate or substance EX with untreated control groups in the River Dale in western Norway. A total of 143 500 smolts were released in 35 release groups in freshwater from 1997 to 2009 and in the fjord system from 2007 to 2009. The adult recaptures declined gradually with release year and reached minimum levels in 2007. This development corresponded with poor marine growth and increased age at maturity of ranched salmon and in three monitored salmon populations and indicated unfavourable conditions in the Norwegian Sea. The recapture rate of treated smolts was significantly higher than the controls in three of the releases performed: the only release in 1997, one of three in 2002 and the only group released in sea water in 2007. The effect of treating the smolts against salmon lice was smaller than the variability in return rates between release groups, and much smaller that variability between release years, but its overall contribution was still significant (*P* < 0.05) and equivalent to an odds ratio of the probability of being recaptured of 1.17 in favour of the treated smolts. Control fish also tended to be smaller as grilse (*P* = 0.057), possibly due to a sublethal effect of salmon lice.

## Introduction

Salmon farming in sea cages has grown to become a large industry since the 1980s. Because of the increase in the numbers of available hosts, the spread of salmon lice larvae is above natural levels in the vicinity of salmon farms (Bjørn & Finstad 2002; Krkosek, Lewis & Volpe 2005; Jansen *et al*. 2012). Salmon lice infestations have been shown to affect the physiology and pathology of salmonids in controlled experiments (Boxaspen 2007; Wagner, Fast & Johnson 2008; Finstad & Bjørn 2011; Finstad *et al*. 2011), and high infestation rates in wild salmonids have been observed (Birkeland & Jakobsen 1997; Finstad *et al*. 2000). However, the ecological consequences of salmon smolts being infested with salmon lice while migrating through regions with salmon aquaculture are poorly understood, and the impact on wild salmon populations has been debated (Costello 2009). Declines in many salmon populations in recent years (Anon 2011a) have contributed to increasing concerns about the possible influence of various anthropogenic factors, such as the reduction in long-term fitness due to enhancement practices (Araki, Cooper & Blouin 2007) and introgression of escaped farmed salmon in wild population (Glover *et al*. 2012) and salmon lice.

Few methods are available for estimating or quantifying the impact of salmon lice on wild populations. One approach has been to treat hatchery-reared smolts against salmon lice and then release them to compare their returns as adults with untreated controls. So far, a total of 37 single release groups have been reported from western Ireland (Jackson *et al*. 2011a,b; Gargan *et al*. 2012) and Norway (Skilbrei & Wennevik 2006b; Hvidsten *et al*. 2007. Krkošek *et al*. (2012) conducted a meta-analysis of all the published data, and estimated the overall effect size (odds ratio) to be 1.29 in favour of the treated smolts. However, the reports demonstrate that the effects of treatment vary greatly across years, release sites and release dates. Most of the groups were moderately or not affected, while the survival of the treated smolts was significantly improved in others. Jackson *et al*. (2011a,b) released smolts in western Ireland from 2001 to 2008 and concluded that salmon lice were of minor importance for survival in the sea. Gargan *et al*. (2012), on the other hand, reported experimental releases from three other locations in western Ireland from 2004 to 2006 and found a much clearer advantage of the treatment for salmon lice. Both Skilbrei & Wennevik (2006b) and Hvidsten *et al*. (2007) found significant differences in survival in one of three smolt releases. The risk of the smolts being infested with salmon lice therefore appears to vary substantially. There is a need for more field experiments to improve estimates of the impact of salmon lice on wild salmon populations, preferably from long-term studies with several releases per year.

Several pioneer farms started salmon production along the coast of western Norway during the 1960s, and the region now hosts a large salmon farming industry (Skilbrei & Wennevik 2006a). It was discovered during the 1990s that salmon lice posed a threat to wild sea trout and salmon smolts in the area (Birkeland & Jakobsen 1997; Heuch *et al*. 2005; Finstad & Bjørn 2011). At the same time, many local salmon populations declined. This development was very dramatic in the River Vosso, long known for its large salmon, which fell to a very low level during the late 1980s and early 1990s (Barlaup 2008). The ecological effects of introgression of escaped farmed salmon (Sægrov *et al*. 1997; Skaala, Wennevik & Glover 2006) and impacts of salmon lice have been proposed as possible causes for this development. Against this background, experimental releases of smolts treated against salmon lice were started in 1997 in the River Dale, which is located in the same fjord and close to the River Vosso. With the exception of 2000, hatchery-reared smolts of River Dale stock have been released every year. This study reports the results of the 35 experimental releases of hatchery-reared smolts from 1997 to 2009. We also tested releases of these smolts on various dates and at different locations in the fjord system and collected wild and stocked smolts in the river for experimental releases in 2004 and 2005. Because of the wide variability in the growth of salmon at sea during this period, we also present data from three wild salmon populations for comparison.

## Materials and methods

Salmon smolts were derived from broodstock collected from the River Dale from 1995 to 2007. The eggs were fertilized in late October or early November. Ten to fourteen family groups were produced each year. The fish were reared in 1 × 1-m indoor tanks under continuous light from first feeding in May. The presmolts were moved between November and early January to between four and eight 2-m-diameter circular tanks under natural photoperiod, which was obtained via a translucent roof above the tanks in which they were kept until time of release.

To ensure thorough mixing of the fish before release, each release group comprised approximately equal numbers of fish from each of the rearing tanks. Fish were anaesthetized (benzocaine, metomidate or MS222) before being tagged with Carlin tags (1997–1999) (Carlin 1955), or adipose fin-clipped and group tagged with sequentially numbered Decimal Coded Wire tags (2001–2009) (Northwest Marine Technology). Approximately 50% of the smolts were treated for salmon lice immediately before release (Tables 1 and 2). Three different treatment methods were used. The prophylactic substance EX (Pharmaq) was used to treat the hatchery smolts in 1997–1999 and 2005, and wild and stocked smolts in 2004 and 2005. Substance EX protects fish from sea lice infection for up to 16 weeks (B. Martinsen, Pharmaq, pers. comm.). The fish were bathed in a solution of 1 mg EX/L-1 for 30 min before release. From 2001 to 2004, and in 2006, the smolts were orally administered 50 μg kg^−1^ emamectin benzoate for 7–8 days prior to release (SLICE®, Schering-Plough Animal Health, 1.5 mm particle size dry feed, manufactured by Skretting AS). From 2007 onwards, 400 μg kg^−1^ emamectin benzoate was administered by intraperitoneal injection (Glover *et al*. 2010) and controls were given sham injection 6–8 days before release.

**Table 1:** Summary data for salmon smolt groups released in River Dale 1997–2009 and their adult recaptures 1998–2011

				Recapture treated		Recapture control				
								
Date released	Number treated	Number control	Smolt length (mm)	*n*	%	*n*	%	Tag	Treatment	*P*
5 May 1997	2975	2978	159	29	0.98	1	0.03	Carlin	EX	<0.001
6 May 1998	2985	2983	172	52	1.74	46	1.54	Carlin	EX	ns
20 May 1999	2940	2959	156	34	1.15	29	0.98	Carlin	EX	ns
23 May 2001	6302	2294	148^a^	47	0.75	25	1.09	Micro	Slice	ns
11 May 2002	1836	1698	158	29	1.58	16	0.94	Micro	Slice	ns
25 May 2002	1771	1761	164	15	0.85	20	1.14	Micro	Slice	ns
7 June 2002	1755	1650		46	2.62	17	1.03	Micro	Slice	<0.001
4 May 2003	2019	2039	150	13	0.64	7	0.34	Micro	Slice	ns
18 May 2003	2023	2082	152	5	0.25	10	0.48	Micro	Slice	ns
2 June 2003	1575	1479	152	8	0.51	4	0.27	Micro	Slice	ns
7 May 2004	1858	1857	147	10	0.54	7	0.38	Micro	Slice	ns
21 May 2004	1866	1859	151	5	0.27	21	1.13	Micro	Slice	<0.005
4 June 2004	1777	1750	156	23	1.27	13	0.74	Micro	Slice	ns
7 May 2005	1750	1750	150	0	0	0	0	Micro	EX	–
20 May 2005	1750	1750	159	0	0	0	0	Micro	EX	–
4 June 2005	1740	1742	181	1	0.06	3	0.17	Micro	EX	ns
7 May 2006	1941	1945	145	1	0.05	6	0.31	Micro	Slice	ns
21 May 2006	1940	1940	157	2	0.10	8	0.41	Micro	Slice	ns
4 June 2006	2262	2271	158	9	0.40	9	0.40	Micro	Slice	ns
9 May 2007	2500	2500	138	0	0	0	0	Micro	Injection	–
23 May 2007	2500	2500	140	0	0	1	0.04	Micro	Injection	–
6 June 2007	2500	2500	143	0	0	0	0	Micro	Injection	–
7 May 2008	1475	1475	164	1	0.07	0	0	Micro	Injection	–
21 May 2008	1500	1500	172	4	0.27	3	0.20	Micro	Injection	ns
3 June 2008	1270	1275	173	0	0	0	0	Micro	Injection	–
3 June 2009	2020	2020	159	6	0.30	8	0.40	Micro	Injection	ns
24 June 2009	1290	1320	167	7	0.54	6	0.45	Micro	Injection	ns
Total	58120	53877		347	0.47	260	0.41			ns

a Measured on 4 April 2001.

**Table 2:** Summary data for salmon smolt groups injected with emamectin benzoate, microtagged, towed and released at different sites in the fjord system 2007–2009 and their adult recaptures 2008–2011. See locations of release sites in Fig. 1

					Recapture treated		Recapture control		
							
Date released	Release site	Number treated	Number control	Smolt length (mm)	*n*	%	*n*	%	*P*
18 June 2007	M5	2110	2200	142	27	1.28	9	0.41	<0.005
20 May 2008	M3	1500	1500	172	22	1.47	17	1.13	ns
26 May 2008	M5	2000	2000	172	16	0.80	21	1.05	ns
13 May 2009	M1	2020	2020	151	12	0.59	19	0.94	ns
27 May 2009	M1	2020	2020	159	38	1.88	44	2.18	ns
13 May 2009	M2	2020	2020	151	47	2.33	40	1.98	ns
27 May 2009	M2	2020	2020	159	28	1.39	18	0.89	ns
30 May 2009	M4	2020	2020	158	37	1.83	32	1.58	ns
Total		15710	15800		227	1.44	200	1.27	ns

More than 6000 wild and stocked smolts were caught in a smolt trap close to the hatchery in 2004 and 2005 (Skilbrei *et al*. 2010) (Table 3). They were treated with substance EX, microtagged and then released in the river. The stocked smolts were siblings of the hatchery-reared smolts, which had previously been released into the river as autumn juveniles.

**Table 3:** Numbers of salmon smolts collected in smolt trap, treated with substance EX or used as controls, microtagged and released during May and June 2004 and 2005. Numbers and percentage returns of adults are also given. *P* is significance level of *G*-test comparing the recapture of treated and control groups

				Recapture treated		Recapture control		
						
Month/year released	Smolt type	Number treated	Number control	*n*	%	*n*	%	*P*
May 2004	Stocked	64	66	0	0	1	1.52	ns
June 2004	Stocked	871	879	2	0.23	2	0.23	ns
Total *2004*	Stocked	935	945	2	0.21	3	0.32	ns
May 2005	Stocked	150	151	0	0	0	0	–
June 2005	Stocked	518	555	0	0	0	0	–
Total *2005*	Stocked	768	706	0	0	0	0	–
May 2004	Wild	392	443	6	1.53	7	1.58	ns
June 2004	Wild	178	107	3	1.69	0	0	ns
May 2004	Wild^a^	405	398	2	0.49	1	0.25	ns
Total *2004*	Wild	975	948	11	1.13	8	0.84	ns
May 2005	Wild	480	476	0	0	1	0.21	ns
June 2005	Wild	312	316	1	0.32	0	0	ns
Total *2005*	Wild	792	792	1	0.13	1	0.13	ns

a Supplementary sample of electrofished smolts.

**Figure 1:**
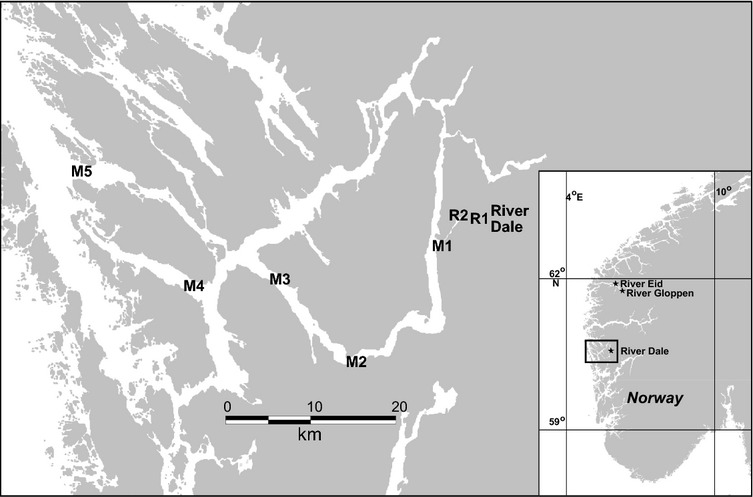
Location of release sites in River Dale (R1 and R2) and in the fjord system (M1, M2, M3, M4 and M5) and River Eid and River Gloppen.

From 1997 to 1999, the smolts were released where the River Dale drains into a 4.3-km-long narrow bay that is connected to the sea, but dominated by fresh water. From 2001 to 2009, the smolts were released directly into the River Dale near the hatchery (Fig 1). There was one release each year in 1997, 1998, 1999 and 2001, three releases every second week from early May to early June in 2002 until 2008, and two releases in 2009 (Table 1). From 2007 on, groups were also transported to the river mouth in a transport tank supplied with oxygen, transferred to a floating transport tank (2007) or net pens (2008 & 2009) by a pipe, and towed to and released at various locations between the river and the coast. One group was released on the coast in 2007, two in the fjord or on the coast in 2008 and five in the fjord in 2009 (Table 2, Fig. 1).

Data from returning adult salmon were derived mainly from angling in the Dale River and from a bag net in the fjord 18 km from the river (69% of recaptures of the 1997–1999 releases and 91% of the 2001–2009 releases). The recovery address was printed on the Carlin tags. Posters were distributed from 2002 onwards to advise the anglers of the microtagged and fin-clipped fish that had been released. A reward of NOK 50 (raised to 100 NOK during the project) was paid to anglers who provided scale samples and the upper jaw (containing the coded wire tag) from adipose fin-clipped salmon. A freezer for storing samples was installed in a room open to the public in the hatchery, which is close to the most productive angling sites.

A wild salmon reference material was built by collecting scales from salmon captured in the Rivers Eid, Gloppen and Dale (see locations in Fig. 1). The scales were read using a microfiche reader printer. Scale characteristics were used to estimate smolt length, smolt age and sea age, and to separate wild from escaped farmed salmon according to the method described by Lund & Hansen (1991). The examination revealed that 970, 3208 and 1397 salmon of wild origin had been sampled in the Dale, Eid and Gloppen, respectively, from 1998 to 2011. From the River Dale, an average of 119 recaptured wild individuals per smolt year class from 1998 to 2004 were analysed, and a mean of 20 wild salmon per year class from 2005 to 2009. In the Rivers Eid and the Gloppen, the numbers of wild salmon from each smolt year class ranged between 94–396 and 49–225 individuals, respectively, from 1998 to 2009.

A 2 × 2 *G*-test (Sokal & Rohlf 1981) was used to test the effect of the sea lice treatment in single release groups. For analyses of multiple release groups, the LOGISTIC procedure of SAS Software Package version 9.1 (SAS Institute) was used to fit generalized linear models (GLM) (McCullagh & Nelder 1989), with a logistic link function to test for differences in the probability of fish being recaptured (binomial response variable) with treatment against sea lice, release year and release date.


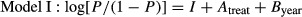



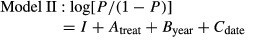



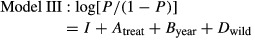


where *P* is the probability of recapture, *I* is the intercept and the categorical parameter *A*_treat_ is the parameter estimate for the effect of the treatment against sea lice (treated or control). The models do not account for the different treatment types. The categorical parameter *B*_year_ and *C*_date_ are the effects of release year and date. Model II was only used when there were several release dates in each single year, that is, from 2002 to 2009. The term *D*_wild_ was included in Model III to compare hatchery-reared and wild smolts released in 2004 and 2005. The GLM module of the statistical package STATISTICA (StatSoft, Release 5.1, v8.0, Statsoft Inc. 2008) was used for the analysis of the weights of the recaptured salmon *(w)* with treatment against sea lice and release year date as category variables: *w* = *I* + *A*_treat_ + *B*_year_.

## Results

**Figure 2:**
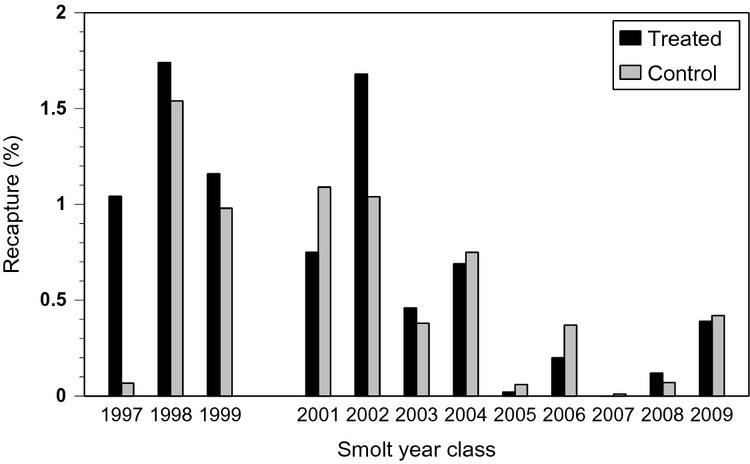
Recapture (%) as adults of hatchery-reared treated (black bars) and control (grey bars) smolts released in River Dale 2001–2009. Note that the figure shows pooled data from multiple releases per year for the period 2002–2009.

In two of the 27 experimental releases of smolts into fresh water, recapture rates of treated adult fish were significantly higher than control recaptures (Table 1); the smolts released in 1997 (*G*-test, *P* < 0.001) and the third group released in 2002 (*G*-test, *P* < 0.001; for detailed analysis of this year class see Skilbrei & Wennevik 2006b). The decline in the recapture rates from 1997 to 2009 (Fig. 2) was reflected in a strong effect of release year (Model I: Wald chi-square: *W* = 270.7, df = 11, *P*_year_ < 0.001), but the treatment also contributed significantly to the variability in the marine survival rates during this period (*W* = 4.3, df = 1, *P*_treat_ < 0.05). An analysis of the years 2002 to 2008, when there were three releases every year at almost fixed dates (Table 1), shows that release year (Model II: *W* = 165.1, df = 6, *P*_year_ < 0.0001) and release date (*W* = 6.2, df = 1, *P*_date_ < 0.05) significantly affected the recapture rates, but sea lice treatment did not (*W* = 1.7, df = 1, *P*_treat_ = 0.19).

**Figure 3:**
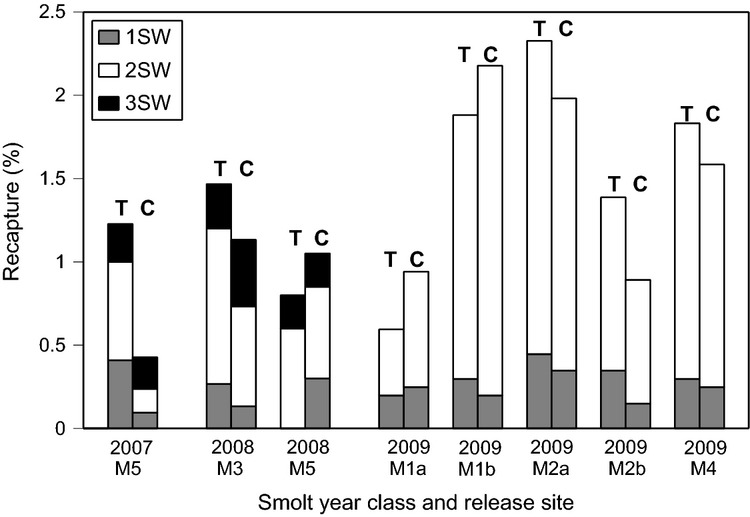
Recapture rates (%) as 1 sea-winter (SW), 2 SW and 3 SW salmon of treated (T) and control (C) smolts released in fjord system 2007–2009 at sites M1–M5 (se Figure 1 for locations), where a and b are the first and the second releases at M1 and M2 in 2009.

The recapture rates of the smolts released in a marine environment were several times as high as those of the smolts released in the river from 2007 to 2009 (Figs. 2 & Figs. 3). The smolts that were towed to the coast in late June 2007 benefited significantly from the sea lice treatment (Fig. 3, *P* < 0.005), but treatment did not affect return rates when all the fjord and coast releases from 2007 to 2009 were taken together (Model II: *W* = 1.8, df = 1, *P*_treat_ = 0.18; *W* = 13.7, df = 2, *P*_year_ < 0.05). From mid- until late-May 2009, the recapture rate of the fish released at release site M1 more than doubled, while the recapture of the M2 release was halved (Fig. 3). There was thus no statistical effect of release date when these four releases were compared (df = 1, *W* = 0.22, *P*_date_ = 0.64).

Overall analysis of all the release groups from 1997 to 2009, irrespective of release site and date, shows that the overall effect of the treatment against sea lice was significant (Model I: *W* = 344.5, df = 11, *P*_year_ < 0.0001; *W* = 5.9, df = 1, *P*_treat_ < 0.05). The recapture rates of all treated and control fish were 0.78 and 0.66%, respectively. The mean yearly recapture rates were 0.82 and 0.68% for the treated and control groups. The odds ratio estimate of the probabilities of recapture of treated versus control fish was 1.17 (Wald 95% confidence limits, 1.03 and 1.32), which imply that a treated fish had 1.17 times the chance of a control fish to be recaptured, but also show a substantial confidence interval. The main reasons for the significant difference were the three single releases with significantly higher returns of treated fish: the 1997 release, the 7 June 2002 release in the River Dale and the 18 June 2007 group released at the coast. When these three release groups are excluded from the analysis, there was no effect of treating the smolts against salmon lice in the remaining 32 release groups (*W* = 0.02, df = 1, *P*_treat_ = 0.89).

Most wild smolts were captured in the smolt trap during May, while stocked smolts were more common in June (Table 3). The total recapture rate of wild smolts released in 2004 was significantly higher than that of the stocked smolts, 1.0 vs. 0.27% (2 × 2 *G*-test, *P* < 0.01), but there was no effect of salmon lice treatment. The recapture rate was zero or close to zero in all groups of wild and stocked smolts in 2005 (Table 3). The total recaptures of wild smolts were somewhat higher than those of hatchery-reared smolts, 0.99 vs. 0.72, and 0.14 vs. 0.04% of the groups released in 2004 and 2005, respectively. The drop from 2004 to 2005 was significant (Model III: *W* = 42.0, df = 1, *P*_year_ < 0.0001), but the differences between wild and hatchery-reared smolts (*W* = 2.4, df = 1, *P*_wild_ = 0.12) and treatment effect (*W* = 0.1, df = 1, *P*_treat_ = 0.80) were not.

**Figure 4:**
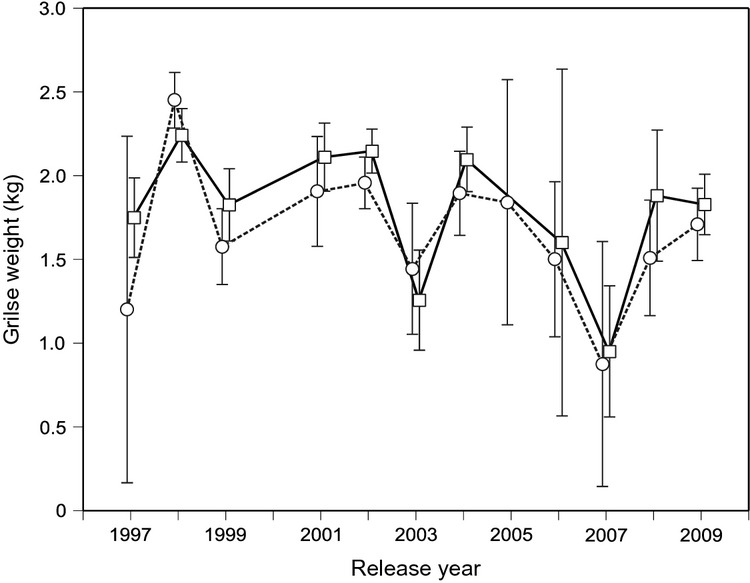
Mean grilse weights of treated smolts released (solid line) and untreated control groups (dashed line) from 1997 to 2009, with 95% confidence intervals.

Generally, grilse (one-sea-winter salmon; 1 SW salmon) weights varied considerably between years (Fig. 4, GLM; df = 10, *F* = 14.1, *P*_year_ < 0.0001). Treatment against salmon lice contributed less to the variability in grilse weight, but on average, the treated fish were 0.1 kg larger than the control fish, 1.79 (SD = 0.05) kg vs. 1.69 (0.05) kg (Fig. 4). This trend was significant for the smolts released in 2002 (details in Skilbrei & Wennevik 2006b) and was very close to significance for the period from 1997 to 2009 (df = 1, *F* = 3.6, *P*_treat_ = 0.057).

**Figure 5:**
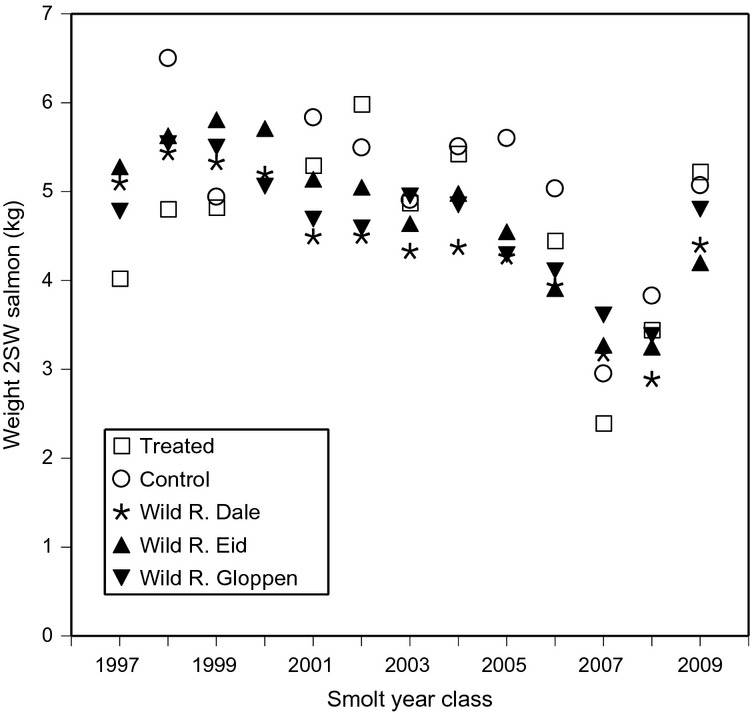
Mean size of two sea-winter treated and control groups and wild fish from the Rivers Dale, Eid and Gloppen.

**Figure 6:**
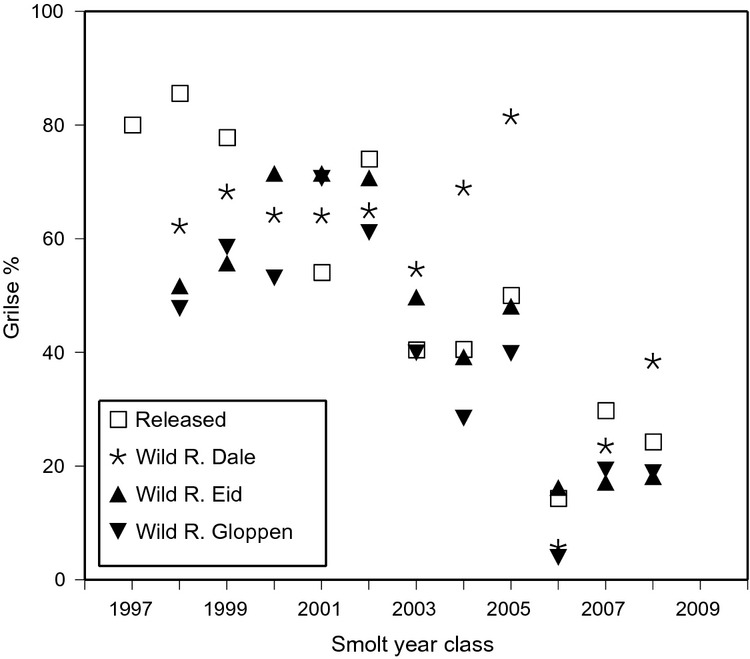
Percentage of grilse relative to multi-sea-winter salmon in adult catches of hatchery-reared smolts released in River Dale 1997–2008 and of wild smolts migrating out of the Rivers Dale, Eid and Gloppen from 1998 to 2008.

Grilse size declined during the experimental period, from about 2 kg for the smolt year classes 1997–2002 to a mean size of less than 1 kg when the 2007 smolts returned. A parallel development was also seen in the 2 SW salmon. The 2007 smolt year class weighed around 3 kg as 2 SW salmon, which is ∼ 60% of the average weights during the first part of the study (Fig. 5). Mean weights of 2 SW salmon then increased sharply towards 5 kg at the end of the study. The relative proportion of fish returning as grilse also declined sharply, from dominance of grilse during the first years to multi-sea-winter salmon being far more numerous during the last years of the study (Fig. 6).

## Discussion

Our study was performed in a region with high production of farmed salmon (Skilbrei & Wennevik 2006a). It was demonstrated that treating Atlantic salmon smolts against salmon lice resulted in a higher percentage of returns than in untreated control groups (odds ratio 1.17), but clear lethal effects of salmon lice were seen in only three of the 35 releases of ranched salmon smolts from 1997 to 2009, and in three of the 12 years examined. These results are similar to those of Jackson *et al*. (2011a,b) who released smolts in Ireland from 2001 to 2008, but show a slightly greater effect of salmon lice. The difference between treated smolts and controls, on the other hand, was less pronounced than the more distinct differences that were observed at three other sites in Ireland in 2004–2006 (Gargan *et al*. 2012), and also lower compared with the meta-analysis of Krkošek *et al*. (2012).

The relatively consistent trend from 1997 to 2009, that treated grilse were ∼ 6% (0.1 kg) heavier than the controls, indicates that the control smolts were normally exposed to sublethal levels of salmon lice. This implies that salmon lice were generally present in the migration route of the smolts, also at times when survival rates did not differ between treated smolts and controls. The suppression of growth was very clear in the smolts released in 2002 (Skilbrei & Wennevik 2006b). Reduced growth and fecundity have also been demonstrated in salmonids infested with sea lice in controlled laboratory experiments (Tveiten *et al*. 2010).

There are several limitations to the methods that may have underscored the effect of salmon lice. A laboratory experiment performed in 2003 concluded that the oral treatment of the Dale hatchery-reared smolts with emamectin benzoate that year resulted in highly variable concentrations of the drug, with some fish receiving only partial dosages, and that the protection lasted for <6 weeks (Skilbrei *et al*. 2008). Because of this uncertainty, intraperitoneal injection of emamectin benzoate was introduced in 2007, which produced a several fold increase in the concentration of the drug in muscle and protection against salmon lice from 2007 of ∼ 9 weeks (Glover *et al*. 2010). Reduced sensitivity of adult salmon lice to emamectin benzoate has been demonstrated in fish farms in western Norway since autumn 2009, and also in Scottish fish farms (Lees *et al*. 2008). The situation is being monitored by the Norwegian authorities, and farmers are still permitted to use Slice® in spring 2012. The use of two drugs and three administration techniques, and the possible development of reduced sensitivity to emamectin benzoate, imply that the duration of the protection against sea lice varied during the experiment. We have not sufficient background information to account for such effects in the models. If we assume that the risk of being infested with salmon lice is highest at the coast in the vicinity of fish farms and lower in open sea, then an important question may be whether the smolts moved through this zone during the first weeks post-releases when they were still protected, regardless of anti-lice treatment method used. The duration of the protection was probably shortest when emamectin benzoate was administered orally (2001–2004 and 2006). The clear differences in survival and marine growth between the treated and control smolts released in 2002 (Skilbrei & Wennevik 2006a) at least indicate that a large portion of the smolts were protected that year.

The release strategy was also improved from 2007 onwards, when groups of smolts were released at various sites along the migration route from the fjord to the coast. The rationale was to increase return rates, which had been low during the previous years, and also to increase the probability that the smolts could reach and pass the outer fjord and coastal areas while still protected against salmon lice. Because of the existence of a surface layer of fresh water in the inner fjord system during spring, the risk of being infested with salmon lice is thought to be low during the first stage of the migration route (Skilbrei 2012). Transfer of the fish to, and releases from, net pens in a higher-salinity environment were beneficial for survival. Reasons for this may have been that the physiological adaptation to sea water, school formation and migratory behaviour may have been stimulated (Skilbrei *et al*. 1994), predation in the river and estuary was avoided, and it may also have been beneficial for the smolts to avoid or reduce their exposure to moderate acid water and aluminium (Al) in the fjord. Watersheds in this area are affected by acid rain mobilizing and transporting aluminium (Al) to the fjord with river water (Barlaup 2008), which have occasionally caused mortality of cultured salmon in the fjord (Bjerknes *et al*. 2003). Under brackish conditions, non-toxic forms of aluminium (Al) present in the fresh water will be transformed to toxic forms of Al, which may precipitate onto fish gills (gill-Al) (Teien, Standring & Salbu 2006). This may impair physiological status, delay migration and reduce the survival of smolts (Kroglund & Finstad 2003; Finstad *et al*. 2007).

The gradual and clear decline in recapture rates of smolts released in the River Dale from ∼ 1–1.7% in 1997–2002 to ∼ 0–0.3% in 2005–2008 may have been caused by unknown factors influencing the survival in the river or the fjord and/or changes in conditions in the ocean. Jackson *et al*. (2011a) observed a similar falling trend in survival rates of Atlantic salmon in Irish release experiments from 2001 to 2008 (from ∼10 to ∼2%), which was parallel for treated and control smolts. Other workers have reported that the survival rates of both wild salmon and released hatchery-reared smolts in the Atlantic Ocean have declined markedly in recent years (Friedland *et al*. 2009; ICES 2010; Otero *et al*. 2011). The reductions in the sizes of the grilse and 2 SW salmon suggest that growth conditions in the sea became poorer. This development was accompanied by the very clear drop in the ratio between grilse and multi-sea-winter salmon during the same period. A lowering of growth rate is generally correlated with increased age at maturity in salmonids (Alm 1959; Taranger *et al*. 2010), but on the other hand, Jonsson & Jonsson (2007) present data showing that the opposite relationship between early marine growth and age at maturity has also been observed. Nevertheless, it seems more likely that the falls in marine survival and growth from 2002 to 2007 were related to changes in the marine ecosystem, rather than by infestation by sea lice. Salmon lice may still have been of importance for marine survival, but its impact is difficult to evaluate during the years when the returns were very low, due to correspondingly low statistical precision. It is also possible that the enhancement practice in River Dale, with releases of juveniles (Skilbrei *et al*. 2010) and smolts, may have influenced its productivity negatively. Due to domestication selection during the captive phase, there is concern that enhancement may reduce the long-term fitness of salmon stocks (Araki *et al*. 2007).

This study has demonstrated that survival rates of released hatchery-reared smolts are highly variable, both within and between years. Environmental fluctuations and more or less stochastic variability in the composition and distribution of predators and food organisms along the migration routes of the smolts have presumably contributed to differences between release groups and demonstrate the need for repetitive releases to characterize the conditions the smolts may experience in the course of a season. The clear differences in survival rates between smolts released in the same fjord ∼ 25 km apart from each other on the same dates in 2009 illustrate the variability and stochastic nature of conditions influencing survival of released smolts. There is an obvious potential to increase smolt survival by developing improved release methods, which could increase statistical accuracy in treatment studies. On the other hand, standardization of release sites and release methods is advisable if the goal is to estimate differences in survival rates at sea in long-term studies. The small and insignificant differences in survival between the hatchery-reared and wild smolts released in 2004 and 2005 were encouraging with regard to the issue of whether results obtained from releases of hatchery-reared smolts are indicative of the marine performance of wild smolts. The low recapture of the stocked smolts, on the other hand, appears to confirm the suggestion of Skilbrei *et al*. (2010) that smolts that originate from stocking of juveniles during the previous autumn migrate several weeks later than wild smolts and suffer from higher marine mortality.

Although other relationships contributed more to the variability in the survival rates of the release groups, salmon lice appeared to impose an average additional marine mortality of ∼ 17% (odds ratio of 1.17 for recapture of treated/control fish). According to the considerations by Norwegian expert groups aiming to quantify the impact of salmon lice, this level of influence would be expected to represent a moderate regulatory effect on a salmon population (Anon 2011b; Taranger *et al*. 2012). While effects of salmon lice were not observed in most of the release groups or in most years, the reduction in survival was dramatic in particular groups, for example the 1997 release, which would have had very serious consequences for the year class if it had been representative for all the smolts that migrated that year. Salmon lice also killed a significant proportion of the smolts in 2002, but in that particular year, the smolt survival was relatively high and returns from the control groups were still acceptable in comparison with other years. The 2007 results exemplify a situation that, under certain circumstances, may represent a clear negative impact of salmon lice, which contributed significantly to increased mortality, while at the same time, oceanic survival and growth rates appeared to be unusually low.

Our data also indicate that non-lethal effects of salmon lice such as reduced growth, and subsequently reduced fecundity, were also a frequent consequence of salmon lice infestation throughout the period under study. It is likely that wild salmon populations in the region were also influenced negatively by salmon lice, but to what extent cannot be easily estimated. Nor is it a simple matter to estimate the population regulatory effects of salmon lice. One reason for this is the apparently stochastic nature of the impact of salmon lice, while control fish performed equally well as treated fish in most release groups and release years, its influence was very clear in the three groups affected. We therefore suggest that evaluations of the risk of negative impact of salmon lice should be linked to the status of the population, which must involve considerations of the combined impacts of salmon lice and other potential stressors. The magnitude of the effects of salmon lice observed in this study may be too small to threaten viable populations, but may give rise to concern for small and vulnerable populations. Large numbers of escaped farmed salmon are found at spawning sites in many Norwegian salmon rivers (Anon 2011a), which may threaten the genetic integrity of populations (Skaala *et al*. 2006). If escaped farmed salmon aggregate in the river while the survival and fecundity of the wild fish are low due to poor oceanic conditions, then the potential impact of salmon lice may increase because of the combined impacts of several negative conditions. A recent study in Norwegian rivers demonstrated that the risk of introgression of fish that is not native to the river, like escaped farmed salmon, is greater in rivers where the number of wild spawners has declined during recent decades; among these is the River Vosso, which is the neighbouring river to the Dale (Glover *et al*. 2012).
